# Platelet distribution width (PDW): A putative marker for threatened preterm labour

**DOI:** 10.12669/pjms.304.4991

**Published:** 2014

**Authors:** Burcu Artunc Ulkumen, Halil Gursoy Pala, Esat Calik, Semra Oruc Koltan

**Affiliations:** 1 Burcu Artunc Ulkumen, Obstetrics and Gynecology Department, Celal Bayar University, Hafsa Sultan Hospital, Uncubozkoy, Manisa-45210, Turkey.; 2 Halil Gursoy Pala, Obstetrics and Gynecology Department, Celal Bayar University, Hafsa Sultan Hospital, Uncubozkoy, Manisa-45210, Turkey.; 3 Esat Calik, Obstetrics and Gynecology Department, Celal Bayar University, Hafsa Sultan Hospital, Uncubozkoy, Manisa-45210, Turkey.; 4 Semra Oruc Koltan, Obstetrics and Gynecology Department, Celal Bayar University, Hafsa Sultan Hospital, Uncubozkoy, Manisa-45210, Turkey.

**Keywords:** Threatened preterm labor, Mean platelet volume, Platelet indices, Platelet distribution width

## Abstract

***Objective: ***To determine the alterations of mean platelet volume (MPV), platelet distribution width (PDW), platelet level and hemoglobin level in pregnancies with threatened preterm labor (TPL).

***Methods: ***The retrospective analysis of 201 pregnant women with threatened preterm labour admitted to our clinic between 2009 and 2013 and 192 healthy pregnancies was conducted. The data regarding the maternal age, hemoglobin level, platelet count, mean platelet volume (MPV), platelet distribution width (PDW) was evaluated.

***Results: ***The mean MPV and hemoglobin levels were significantly lower in TPL group (p=0.001 and p=0.01, respectively). PDW levels were significantly higher in TPL group (p=0.05). (p=0.01). Regarding the platelet count, there was no statistically significant difference between the TPL and control groups. ROC curve analysis for PDW revealed an area under curve (AUC) 66.8%. By using a cut-off value 16.15 for PDW, sensitivity was 76.1% and specificity was 43.5% for TPL.

***Conclusion: ***MPV seems to be lower in threatened preterm deliveries, whereas PDW levels were increased suggesting the possible high grade inflammation and platelet activation in the pathology. Anemia occurs more frequently in threatened preterm delivery. Increased PDW levels especially > 16.15 may alert the obstetrician for the risk of the preterm delivery. However, further studies are needed to state the usefulness of the platelet indices in the diagnosis and clinical follow-up of preterm labor.

## INTRODUCTION

Preterm delivery involves births before 37 gestational weeks and accounts for approximately 10% of all deliveries.^1^ Preterm birth especially before 32 gestational weeks is associated with high rates of neonatal mortality and morbidity also with long term sequelae.^2^ Approximately 28% of all neonatal deaths occuring during the first week of life can be attributed to preterm birth.^3^ Inflammation, infection, demographic factors, genetic factors, stress may play role in the etio-pathogenesis of preterm birth.^1^

Recent studies have highlighted the role of platelets and platelet-derived agents in thrombosis, angiogenesis, inflammation and immunity.^4^ Platelet activation occurs primarily in the process of hemostasis if any damage to blood vessel takes place. Besides, platelet activation also occurs in acute and chronic inflammatory response process. It is well known that in preterm delivery, some inflammatory cytokines are increased both in maternal-fetal interface and systemic circulation.^5^ However, there was no much data regarding the relation between platelet activation and preterm labor. Only published study by Erez et al suggested that there was an increased thrombin activation in preterm labor which may result in platelet activation^6^. The platelet causes some morphological alterations in the platelets: they seem larger by becoming spherical in shape and by forming pseudopodia. As a result, platelets with enhanced number and size of pseudopodia will be different in size leading alterations in PDW and MPV.^7^

Our objective in this study was to evaluate the alterations of platelet indices and investigate whether they can be used as a predictor marker for threatened preterm birth.

To the best of our knowledge, alterations in MPV and PDW in pregnancies with threatened preterm labor (TPL) have not been investigated before. The objective of this unique study was to evaluate whether MPV and PDW were changed in pregnancies with TPL.

## METHODS

This retrospective study was carried out at the department of obstetrics and gynecology of a tertiary center and was approved by the Institutional Ethics Committee. The data of 201 pregnant women with threatened preterm labour between 2009 and 2013 was analyzed. The control group matched for maternal age, parity and gestational week consisted of 192 healthy pregnant women during their early third trimester. The data regarding the maternal age, hemoglobin level (g/dl), platelet count (count/mm^3^), mean platelet volume (MPV), platelet distribution width (PDW) were evaluated. Patients with premature preterm rupture of membranes (PPROM), proven or suspected maternal or fetal infection were excluded from the study. Pregnancies with spontaneous TPL were included for the study group. Patients with chronic inflammatory diseases, i.e. connective tissue disorders such as systemic lupus erythematosus, rheumatoid arthritis, vasculitis, renal or hepatic insufficiency, hemoglobinopathies, diabetes mellitus, hypertensive disorders, previous myocardial infarction, previous thrombosis history were excluded from the study.

The blood samples were taken just after the admission to our clinic. All blood samples were collected in tubes with EDTA (potassium ethylenediaminetetraacetate) which served as the anticoagulant agent. All the blood samples were analyzed by the hematology analyzer within two hours of sampling (MINDRAY BC-6800).

The data was analyzed using the Statistical Package for Social Sciences (SPSS) software version 20. The results were expressed in terms of mean ± standard deviation (SD). A two-tailed p value <0.05 was regarded as statistically significant for all comparisons. T-test was used to compare the different groups. ROC analysis was performed in order to investigate the diagnostic performance of any marker.

## RESULTS

**Fig.1:**
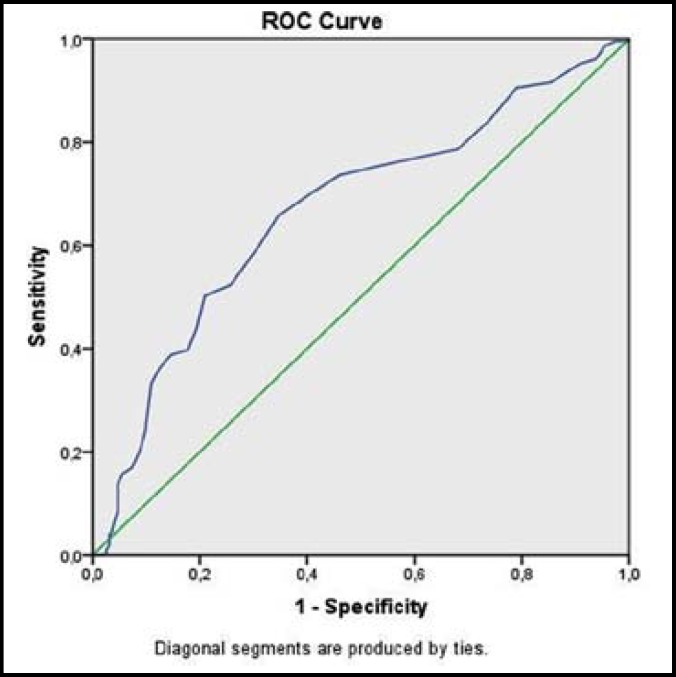
ROC analysis of PDW for screening TPL

The mean age of TPL group was 26.45.9 and the mean age of the control group was 27.55.3 (p > 0.05). The mean gestational week of TPL group was 32.55.8 and the mean gestational week of the control group was 30.45.3 (p > 0.05). The main demographic data about gravida, parity, gestational week and maternal age is shown in Table-I. Platelet values, mean platelet volume and platelet distribution width levels of the study and control groups are shown in Table-II. The mean hemoglobin levels were 9.83.8 g/dl and 14.51.9 g/dl in TPL and control group, respectively (p=0.01). The mean hemoglobin levels were significantly lower in TPL group. The mean platelet levels were 218.24067.990 /mm^3 ^and 223.87068.190 /mm^3^, MPV levels were 9.23±1.38 and 9.71±1.47, PDW levels were 16.78±0.96 and 15.75±10.25 in TPL and in control groups, respectively (p= 0.414, 0.01, 0.05, respectively). Regarding the platelet count there was no statistically significant difference between the TPL and control groups. However, MPV levels were significantly lower in TPL group (p=0.01); whereas PDW levels were significantly higher in TPL group (p=0.05) (ROC curve analysis for PDW revealed an area under curve (AUC) 66.8%. By using a cut-off value 16.15 for PDW, sensitivity was 76.1% and specificity was 43.5% for TPL (Fig.1).

## DISCUSSION

Preterm delivery is an important cause of neonatal mortality and morbidity.^1^^,^^2^ Approximately, 1.1 million infants die due to prematurity annually. The survivors come up against both short- and long-term sequelae, such as respiratory and neurologic deficiencies.^8^ In the last twenty years, the preterm delivery incidence has increased worldwide.^8^^,^^9^ Spontaneous preterm delivery accounts for 45% of all preterm births. Preterm delivery due to preterm premature rupture of membranes (PPROM) and due to maternal or fetal infections accounts for 25% and 30%, respectively. The possible pathologic process for preterm delivery may include inflammation, utero-placental ischemia, immunologic reactions and maternal stress.^9^

Complete blood count (CBC) is routinely checked for all pregnant women. Platelet count and platelet indices such as MPV and PDW are parameters of the routinely checked CBC. Being natural sources of growth factors like insulin-like growth factor 1 (IGF-1), platelet-derived growth factor (PDGF), vascular endothelial growth factor (VEGF), or transforming growth factor β (TGF-β) caused the platelets to have important role in different processes such as inflammation, angiogenesis, repair and regeneration of the tissues.^10^ In TPL patients, due to inflammation and possible ongoing ischemia process, we hypothesized that platelet activation indices like MPV and PDW should be altered than the controls. Previous studies suggested that in inflammation via some cytokines, the platelets size and volume alter differently: in low grade inflammatory disorders, by the involvement of the large platelets in thrombi, MPV values may increase. On the other hand, in high grade inflammatory conditions, the consumption of the large platelets at the inflammation site cause a decrease in MPV levels.^11^ It is well known that pregnancy itself and also labor causes platelet activation.^12^^-^^14^ Platelet indices vary also depending on the gestational week. In general, dilutional thrombocytopenia exists with an compensatory increase in MPV and PDW levels during the pregnancy.^15^^,^^16^ Cardiovascular risk factors like smoking status, hypertension, dyslipidemia, diabetes also affect the size of platelets.^17^ In our study, TPL and control group were at 32.55.8 and 30.42.4 gestational week, respectively (p>0.05). This excludes the effect of the gestational week on platelet indices, as they are approximately in the similar trimester. We think that lower MPV levels in our study may be the result of the possible high grade inflammation in the etiology of TPL. In a previous study by Saleh et al, it was shown that mean platelet factor-4 and beta-thromboglobulin plasma concentrations were higher in maternal circulation with preterm labor compared with levels of term labor.^18^ In agreement with the suggestion of platelet activation in preterm labor, a recent study showed that maternal plasma sCD40L levels were higher in preterm labor compared with the concentrations of term labor.^6^

**Table-I:** Demographic features of the TPL and control group

	***TPL group*** ***n:201***	***Control group*** ***n:192***	***p***
Mean age (mean±SD)	26.45.9	27.55.3	0.056
Gravida (mean±SD)	1.81.3	2.22.5	0.079
Parity (mean±SD)	0.560.9	0.70.9	0.079
Abortus (mean±SD)	0.30.7	0.40.9	0.325
Mean gestational week (mean±SD)	32.55.8	30.42.4	0.219

**Table-II:** Peripheral blood changes in TPL and control group

	***TPL group*** ***n:201***	***Control group*** ***n:192***	***p***
Hemoglobin (g/dl) (mean±SD)	9.863.8	14.51.8	0.01*
Hematocrite (mean±SD)	29.110.3	33.59.5	0.005*
WBC (/mm^3^) (mean±SD)	3503.64543.6	64165009.1	0.02*
Platelet count (/mm^3^) (mean±SD)	218.24067.990	22368.19	0.414
MPV (fL) (mean±SD)	9.231.38	9.711.47	0.01*
PDW (mean±SD)	16.780.96	15.75±10.25	0.05*

* *p<0.05: statistically significant*.

All blood samples in our study were collected in tubes containing potassium ethylenediaminetetraacetate (EDTA) as the anticoagulant agent and they were analyzed in two hours following the sampling. Previous studies mentioned some difficulties in interpreting the results about the platelet indices.^7^^,^^8^ The fact that MPV and PDW levels change in a time-dependent manner lead to some different results according to the time period between the blood sampling and sample analyzing. Furthermore, the use of different anticoagulant agents in the blood tube also affect the platelet indices.^7^ These above-mentioned factors may be the cause of different comments in previous studies about platelet indices.^11^^-^^16^ However, previous studies showed that MPV values are not affected if the measurement is within one hour of blood sampling.^19^ The limitation of our study is that we had no control on the time period between sampling and measurement as this study was conducted retrospectively. However, in contrast to the expected time-dependent increase in MPV and decrease in PDW, we noticed that MPV levels were significantly lower and PDW levels were significantly higher in the TPL group, which created a hope for us that decreasing MPV and increasing PDW levels may predict TPL. ROC curve analysis for PDW levels revealed that PDW levels may be used for discriminating high risk population for preterm labour. By using a cut-off value 16.15 for PDW, sensitivity was 76.1% and specificity was 43.5% for TPL.

In our study, we found lower hemoglobin levels in TPL group. The association between maternal anemia and preterm delivery is controversial. Several studies have showed that maternal anemia was associated with preterm labor,^20^^,^^21^ whereas some studies found no correlation between maternal anemia and poor pregnancy outcomes including preterm delivery.^22^^,^^23^

As a result, our preliminary findings showed that PDW levels > 16.15 may predict TPL with 76.1% sensitivity and 43.5% specificity. Further prospective studies with larger sample size are needed to confirm this finding.

## Authors Contribution

Burcu Artunc Ulkumen designed the study and did statistical analysis & editing of manuscript

Halil Gursoy Pala was involved in designing and manuscript writing

Esat Calik did data collection.

Semra Oruc Koltan conceived the project, did review and final approval of manuscript
